# T-Bet and Eomes Regulate the Balance between the Effector/Central Memory T Cells versus Memory Stem Like T Cells

**DOI:** 10.1371/journal.pone.0067401

**Published:** 2013-06-27

**Authors:** Gang Li, Qianting Yang, Yibei Zhu, Hong-Rui Wang, Xinchun Chen, Xueguang Zhang, Binfeng Lu

**Affiliations:** 1 Stem Cell Research Laboratory of Jiangsu Province, Jiangsu Institute of Clinical Immunology, Institute of Medical Biotechnology, Soochow University, Suzhou, Jiangsu, China; 2 Department of Immunology, University of Pittsburgh School of Medicine, Pittsburgh, Pennsylvania, United States of America; 3 Guangdong Key Lab for Emerging Infectious Disease, Shenzhen Key Lab of Infection and Immunity, Third People’s Hospital, Guangdong Medical College, Shenzhen, Guangdong, China; 4 Department of Immunology, Medical College of Soochow University, Suzhou, Jiangsu, China; 5 State Key Laboratory of Cellular Stress Biology, School of Life Sciences, Xiamen University, Xiamen, Fujian, China; University of California, San Francisco, United States of America

## Abstract

Memory T cells are composed of effector, central, and memory stem cells. Previous studies have implicated that both T-bet and Eomes are involved in the generation of effector and central memory CD8 T cells. The exact role of these transcription factors in shaping the memory T cell pool is not well understood, particularly with memory stem T cells. Here, we demonstrate that both T-bet or Eomes are required for elimination of established tumors by adoptively transferred CD8 T cells. We also examined the role of T-bet and Eomes in the generation of tumor-specific memory T cell subsets upon adoptive transfer. We showed that combined T-bet and Eomes deficiency resulted in a severe reduction in the number of effector/central memory T cells but an increase in the percentage of CD62L^high^CD44^low^ Sca-1^+^ T cells which were similar to the phenotype of memory stem T cells. Despite preserving large numbers of phenotypic memory stem T cells, the lack of both of T-bet and Eomes resulted in a profound defect in antitumor memory responses, suggesting T-bet and Eomes are crucial for the antitumor function of these memory T cells. Our study establishes that T-bet and Eomes cooperate to promote the phenotype of effector/central memory CD8 T cell versus that of memory stem like T cells.

## Introduction

Tumor growth can elicit type 1 cellular immune responses that limit cancer progression. Ample clinical evidence shows that longer survival of cancer patients is associated with increased expression of genes characteristic of type 1 effector T cells, in particular master transcription regulators T-bet and Eomes. [1]–[5] In T cells, T-bet and Eomes are regulated by cytokines with divergent functions and therefore have overlapping as well as distinct functions [6]–[11]. IL-12 and IFN-γ drive T-bet expression, [12], [13] and IL-2 promotes Eomes expression. [7], [14], [15] T-bet and Eomes play an additive role in driving IFN-γ production and cytotoxic activities of effector CD8 T cells in vitro. [8], 16 T-bet and Eomes also coordinately promote T cell migration to inflamed tissues by inducing chemokine receptors. [16], [17] In addition, T-bet and Eomes control the expression of CD122 and are required for maintenance of IL-15-dependent memory CD8 T cells. [10], [11] High T-bet expression promotes short-lived effector CD8 T cells, whereas low T-bet expression promotes long-lived memory cells. [18], [6], [11], [19] Thus, T-bet and Eomes are important for both function and homeostasis of effector and memory T cells. However, the role of T-bet and Eomes in the setting of memory T cell responses to tumor antigens is unknown.

The memory T cells have been typically divided into two main subsets based on the expression of the lymph node homing molecules CD62L and CCR7. [20] Central memory T cells (T_CM_) express high levels of CD62L and CCR7, whereas effector memory T cells (T_EM_) express low levels of CD62L and CCR7. Recent studies demonstrated the existence of a new population of memory T cells designated T memory stem cells (T_SCM_) [21] [22]. T_SCM_ are CD44^low^ CD62L^high^, a phenotype similar to those of naïve T cells [21]. Nevertheless, they differ from naïve cells by expressing stem cell antigen–1 (Sca-1) and proliferate vigorously upon restimulation with its antigenic peptide [21] [23] [22]. Although T-bet and Eomes are known to be involved in both function and homeostasis of effector and memory T cells, their role in T_SCM_ is not studied.

Adoptive T cell therapy has become increasingly appreciated as a feasible therapeutic approach for human cancer. The infused tumor antigen-specific T cells are believed to adopt multiple effector and memory T cell fates in the host. Since T-bet and Eomes are master transcriptional factors for CD8 T cells, we studied their individual and collective roles in determining the phenotype and function of adoptively transferred T cells. We demonstrated that T-bet and Eomes play a synergistic role during the effector phase of an antitumor immunity. In addition, both T-bet and Eomes are required for the maintenance of effector and central memory CD8^+^ T cells. Interestingly, we found that the absence of both T-bet and Eomes resulted in a T cell population dominated by phenotypically-defined T_SCM_. Our study establishes that the T-bet and Eomes transcriptional unit regulates the balance between effector/central memory T cells and T_SCM_.

## Methods

### Mice

Generation of CD4-cre Eomes fl/fl (EKO) and T-bet−/− CD4-cre Eomes fl/fl (DKO) mice has been described [16]. Pmel-1 TCR transgenic mice were purchased from the Jackson Laboratory and bred with TKO (the Jackson Laboratory), EKO, and DKO mice. B6-LY5.2/Cr mice were purchased from Frederick National Lab. All animal experiments have been approved by IACUC of University of Pittsburgh and IACUC of Soochow University.

### Adoptive T cell Therapy

B6-LY5.2/Cr mice were challenged with 3×10^5^ B16F0 cells *i.d.* 6 days later, mice were irradiated at 500 rad. On day 7, the mice were adoptively transferred with 5×10^5^ WT, T-bet−/−, Eomes−/−, or T-bet/Eomes DKO pmel-1 T cells which have been cultured in Th1 condition for 3 days. Tumor growth was monitored every two days.

To examine the prophylactic function of adoptive transfer T cells, B6-LY5.2/Cr mice were irradiated at 500 rad. 24 h post irradiation, the mice were adoptively transferred with 4×10^6^ WT, T-bet−/−, Eomes−/−, or T-bet/Eomes DKO pmel-1 T cells which have been cultured in Th1 conditions for 4 days. 1 month later, the recipient mice were challenged with 2×10^5^ B16F0 cells *i.d.*

### T Cell Culture

For generation of effector pmel-1 T cells, spleen and lymph node were collected from C57BL/6 WT, TKO, EKO, and T/E DKO pmel-1 TCR transgenic mice. CD8^+^ T cells were purified using anti-CD8 magnetic beads (Miltenyi Biotech). Cells were stimulated with irradiated T cell free APC, 1 µM mgp100_25–33_ (EGSRNQDWL), IL-2 (20 units/ml) and IL-12 (3.4 ng/ml) plus anti–IL-4 (10 µg/ml, clone 11B11) for Tc1 for 72 h. APCs were prepared from splenocytes that are depleted of T cells and irradiated by 3000 rad.

### Flow Cytometric Analysis

CD4 (clone GK1.5), CD8 (clone 53-6.7), CD44 (clone IM7), CD122 (clone 5H4), CD62L (clone MEL-14), CD127 (clone A7R34), CD45.1 (clone A20) and CD45.2. (clone 104) were purchased from eBioscience (San Diego, CA). Flow cytometric analysis was performed using a FACS flow cytometer (BD Biosciences, San Jose, CA).

For intracellular cytokine staining, harvested cells were stimulated with PMA (10 ng/ml) and ionomycin (1 µg/ml) for 4 h and incubated for the last 1 h with brefeldin A (10 µg/ml). Cells were subjected to intracellular cytokine analysis with anti–IL-17 (eBio17B7; eBioscience) and anti–IFN-γ Ab (clone XMG1.2; eBioscience).

### Harvest of Tumor-infiltrating Lymphocytes

Tumor masses were removed, minced, and digested with collagenase and hyaluronidase digestion solution (2.5 mg/ml collagenase I, 1 mg/ml collagenase IV, 0.25 mg/ml hyaluronidase IV-S, 300 µg/ml DNase I, and 0.01% HEPES in RPMI 1640 medium) at 37°C for 2 h. The pieces were then gently pressed between the frosted edges of two sterile glass slides, and the cell suspension was filtered through a 40-µm cell strainer (BD Biosciences). Tumor-infiltrating lymphocytes (TILs) were further purified by using the gradient as per manufacturer protocol, washed, and re-suspended in HBSS for analysis.

## Results

### T-bet and Eomes are Required for Adoptive Tumor Immunotherapy in Mice

**Figure 1:**
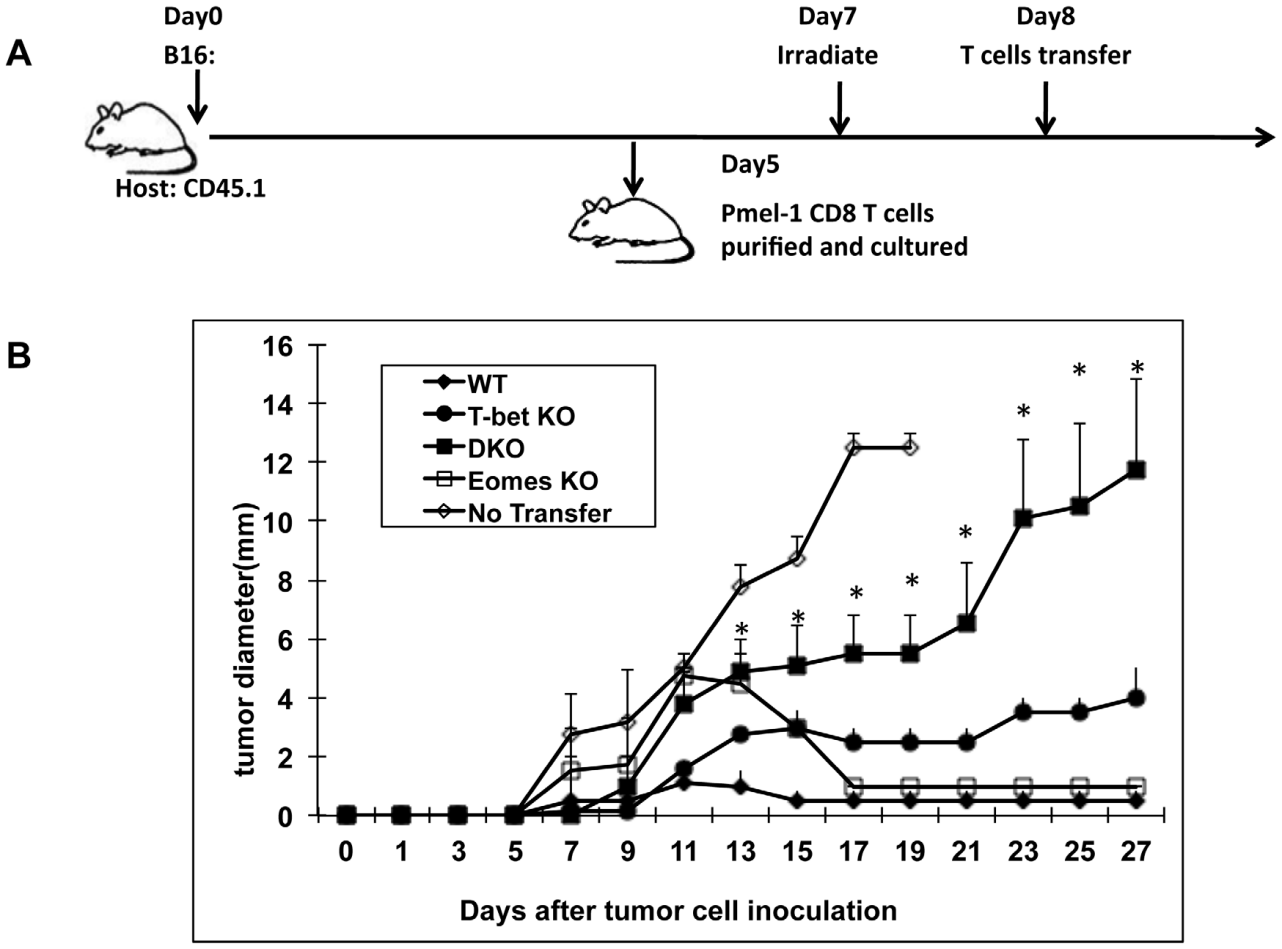
Adoptive immune therapy in mice requires both T-bet and Eomes. B6-LY5.2/Cr mice were challenged with 3×10^5^ B16F0 cells *i.d.* 6 days later, mice were irradiated at 500 rad. On day 7, the mice were adoptively transferred with 5×10^5^ WT, T-bet−/−, Eomes−/−, or T-bet/Eomes DKO pmel-1 T cells which have been cultured in Th1 condition for 3 days. Tumor growth was monitored every two days. 5 mice were in each group. The no-transfer group did not receive any T cells. *P<0.05**,** determined by Mann-Whitney Test comparing recipients infused with DKO and WT T cells.

Adoptive T cell therapy is a promising strategy for the treatment of cancer. [24], [25] To begin to dissect the genetic program regulating the function of adoptively transferred T cells in tumor-bearing hosts, we first focused on T-bet and Eomes.[1]–[5] To generate tumor-reactive CD8^+^ T cells, we used T cells from WT, T-bet−/− (TKO), Eomes−/− (EKO), and T-bet−/− Eomes−/− (DKO) pmel-1 T cell receptor transgenic mice. [26] The number of naïve pmel-1 T cells was comparable among all strains. We stimulated pmel-1 naïve T cells in vitro in Th1 conditions for three days to generate effector T cells specific for melanoma antigen (Ag) gp100. At this time point, most of the T cells had proliferated as determined by carboxyfluorescein diacetate succinimidyl ester (CSFE) staining and there was no difference in proliferation between the strains (data not shown), consistent with our previous results and suggesting that T-bet and Eomes were not required for clonal expansion of CD8 T cells [27]. In addition, the number of live cells was similar among WT, TKO, EKO, and DKO pmel-1 T cells after 72 h in culture (data not shown). We then infused the effector T cells to B16 melanoma-bearing mice that had been non-lethally irradiated 24 h before. Tumor growth was inhibited in recipient mice adoptively transferred with WT pmel-1 T cells (Figure 1). Similarly, tumor growth was inhibited in mice infused with either TKO or EKO pmel-1 T cells, suggesting T-bet and Eomes were individually non-essential (Figure 1). In contrast, adoptively transferring DKO pmel-1 T cells failed to inhibit the tumor growth (Figure 1). These data demonstrated that both T-bet and Eomes were required for adoptive therapy of cancer.

### T-bet and Eomes Control the Number, Trafficking and Effector Function of Adoptively Transferred T cells

**Figure 2:**
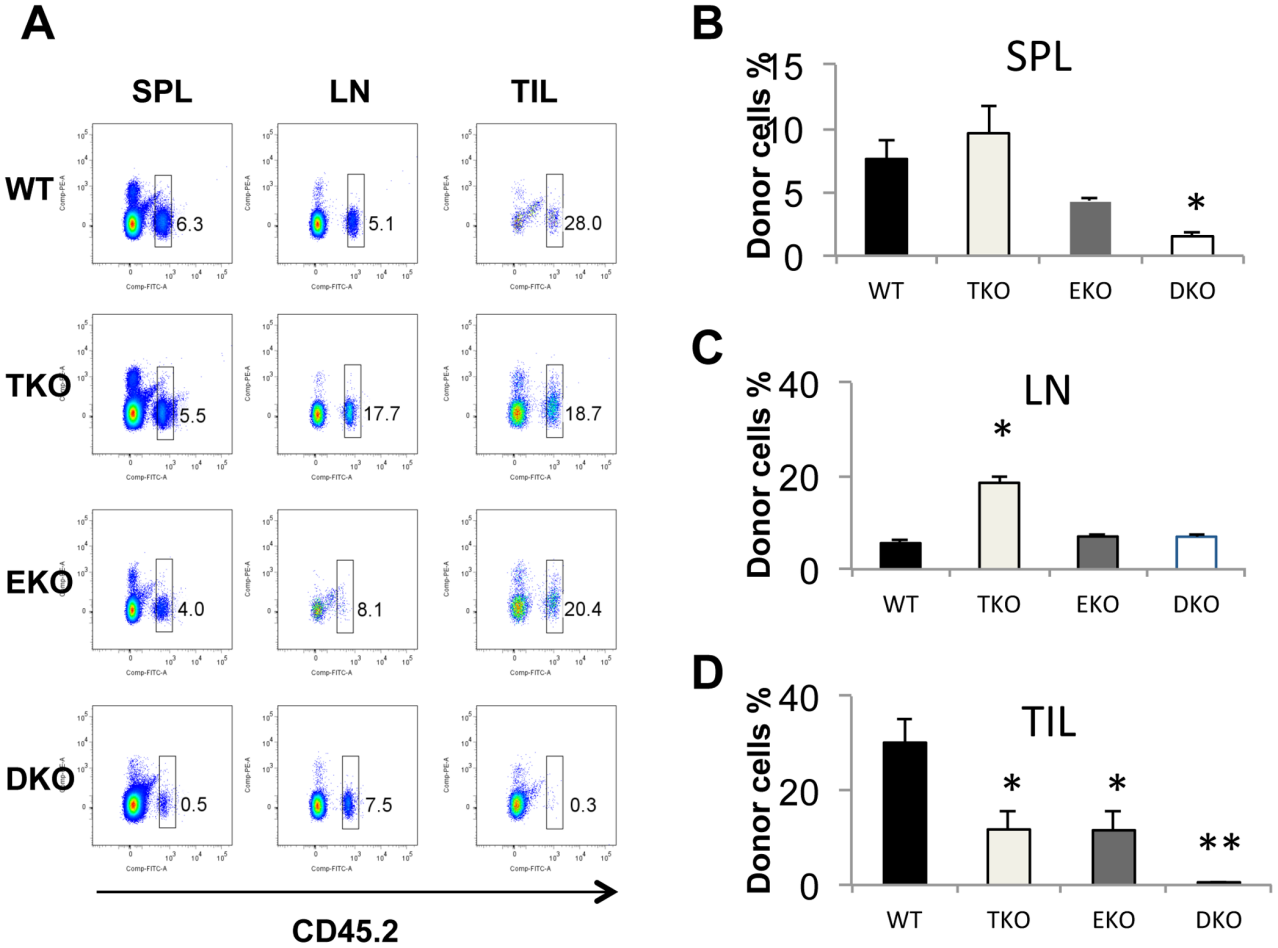
T-bet and Eomes regulate tissue distribution and survival of the adoptive transferred T cells in tumor-bearing recipient mice. B6-LY5.2/Cr mice were challenged with 3×10^5^ B16F0 cells *i.d.* 6 days later, mice were irradiated at 500 rad. On day 7, the mice were adoptively transferred with 5×10^5^ WT, T-bet−/−, Eomes−/−, or T-bet/Eomes DKO pmel-1 T cells which have been cultured in Th1 condition for 3 days. (A) Splenocytes (SPL), lymph node (LN) and tumor infiltrating lymphocytes (TILs) were harvested from tumor-bearing mice 20 days after T cell infusion. The percentage of donor cells (CD45.2^+^) cells was determined by flow cytometry and the representative dot plots were shown. (B) The average percentage of donor cells (CD45.2^+^) in spleen was shown (N = 4 for each genotype). (C) The average percentage of donor cells (CD45.2^+^) in lymph nodes was shown (N = 4 for each genotype). (D) The average percentage of donor cells (CD45.2^+^) in tumor was shown (N = 4 for each genotype). Data are presented as mean ± SEM of four mice for each genotype. *P<0.05, **P<0.01 by Student’s t-test.

Since T-bet and Eomes are induced by different signals, [27] it is conceivable that they might regulate different functions of the adoptively transferred T cells in vivo. We first examined the frequency of adoptively transferred pmel-1 T cells in secondary lymphoid organs and tumor sites on approximately 20 days post infusion. The purity of infused pmel-1 T cells were about 95% CD8^+^ Vβ13^+^ (data not shown), and CD44 was highly upregulated in pmel-1 T cells regardless of their genotype (Figure S1 in file S1). The donor T cells can be distinguished from endogenous T cells by their distinctive CD45 alleles. We confirmed that majority of CD45.2^+^ cells were CD8^+^Vβ13^+^ upon infusion (Figure S2 file S1). Thirty days after infusion, about 7% of the lymphocytes in the spleen were donor CD45.2^+^ WT T cells (Figure 2 A and B). Similar percentages of infused T cells were found in mice receiving TKO or EKO T cells (Figure 2 A and B). In contrast, only 1% of donor DKO pmel-1 T cells could be detected at this time point (Figure 2A), and this was statistically significant (Figure 2B). In lymph nodes of the recipient mice, about 5% of the lymphocytes were adoptively transferred WT T cells. Similar percentages of EKO adoptively transferred T cells were observed in the lymph node (Figure 2 A and C). In contrast, the percentage of adoptively transferred TKO T cells was increased significantly to about 20% in lymph nodes (Figure 2 A and C). Surprisingly, the percentage of DKO T cells was similar to that of WT T cells in the lymph nodes (Figure 2 A and C). These data suggest that T-bet and Eomes might play different roles in the survival and trafficking of adoptively transferred effector T cells in secondary lymphoid organs.

The antitumor effect of adoptively transferred T cells is ultimately determined by their ability to migrate to and carry out effector functions within the tumor. We therefore examined the frequency of the adoptively transferred pmel-1 T cells within the B16 tumor. Approximately 30% of tumor infiltrating lymphocytes (TIL) were WT pmel-1 T cells (Figure 2 A and D). Either T-bet or Eomes deficiency resulted in a modest but statistically significant reduction in the percentage of the donor T cells among the TILs (Figure 2 A and D). Strikingly, T-bet and Eomes double deficiency greatly reduced the number of donor T cells in the tumor tissues (Figure 2 A and B). These data demonstrated that T-bet and Eomes synergistically control T cell persistence within the tumor. This is likely due to the combined effect of lack of expression of chemokine receptors such as CXCR3 as we have reported in CD8 T cells previously [16], reduced survival due to reduced CD122 [10], and diminished effector function such as IFN-γ production and cytolytic activities.

**Figure 3:**
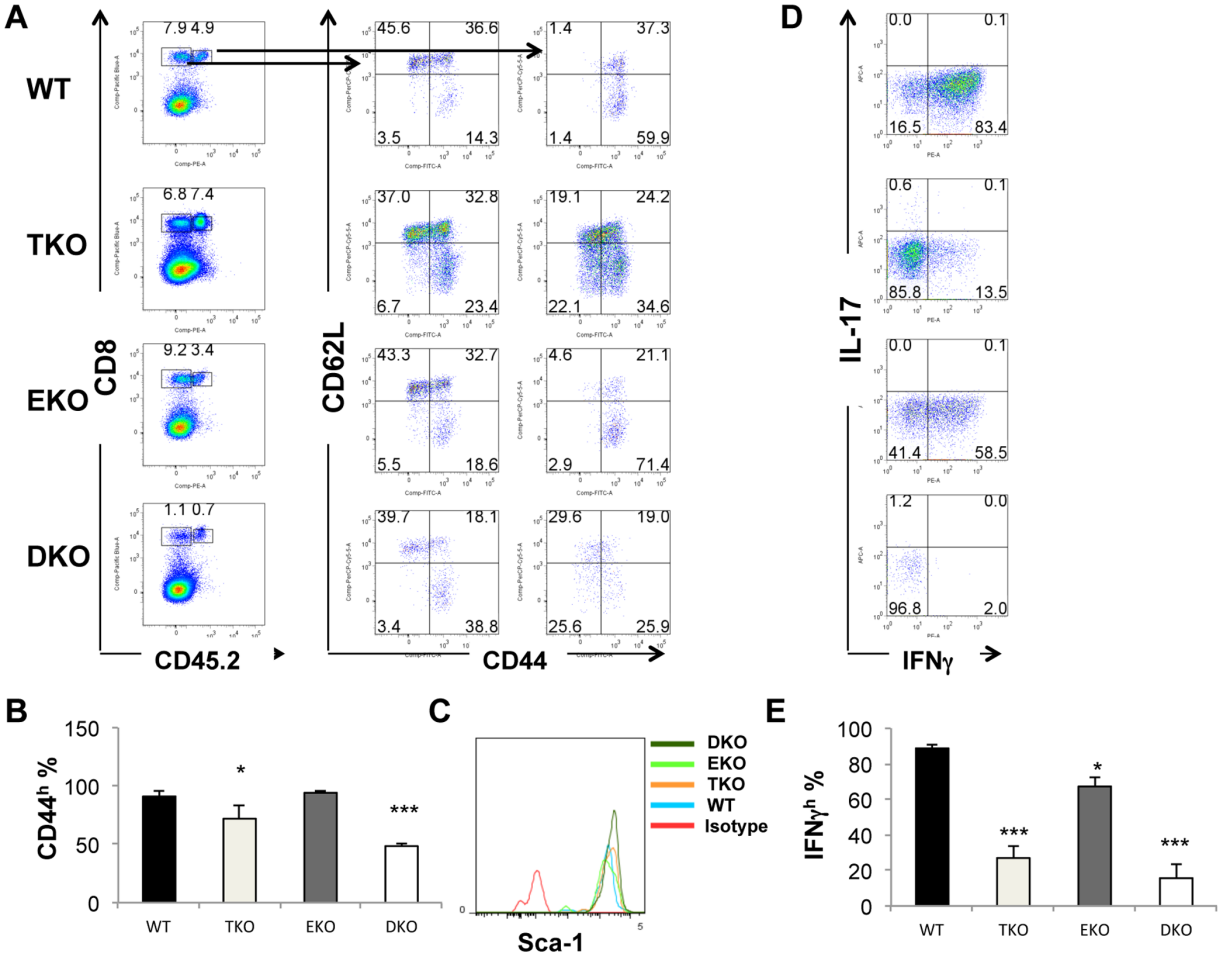
T-bet and Eomes regulate the surface marker and function and adoptive transferred T cells in tumor-bearing mice. Pmel-1 T cells were adoptively transferred to tumor-bearing B6-LY5.2/Cr mice as in figure 1. 20 days post T cell infusion, spleen cells were harvested and analyzed. (A) Dot plots show percentage of CD44^hi^CD62L^hi^, CD44^hi^CD62L^lo^ and CD44^lo^CD62L^hi^ T cells in within donor (CD45.2^+^) and recipient (CD45.2^−^) CD8^+^ T cells. (B) The average percentage of donor cells (CD45.2^+^) CD44^+^ CD8^+^ T cells in the spleen was shown (N = 4 for each genotype). (C) Sca-1 levels in the adoptive transferred CD8 T cells were shown. (D). Splenocytes were harvested from different recipient tumor-bearing mice, were subsequently stimulated with PMA and ionomycin for 4 hours and incubated for the last 1 hour with brefeldin A. The percentage of donor pmel-1 CD8^+^ T cells producing IFN-γ and IL-17 were examined. (E) The average percentage of IFN-γ^+^ donor cells CD8^+^ T cells in the spleen as shown in (D) are shown (N = 4 for each genotype). Data are presented as mean ± SEM of four mice for each genotype. *P<0.05, ***P<0.001 by Student’s t-test.

To further characterize adoptively transferred effector T cells, we examined the surface markers such as CD44 and CD62L that are characteristic of naïve and memory T cell subsets. The expression of CD44 and CD62L on the endogenous CD8 T cells in spleens was not changed in all recipient mice regardless of the genotype of the infused T cells (Figure 3A). Close to 100% of the WT and EKO donor T cells were CD44^high^. About two thirds of the WT and EKO CD44^+^ T cells were CD62L^low^, characteristic of effector cells, and one third were CD62L^high^, typical of central memory T cells. Therefore, the persistent WT and EKO T cells consisted mainly of phenotypic central and effector memory T cells. In contrast, the level of CD44 on the adoptively transferred TKO T cells was modestly reduced (Figure 3 A and B). And a population of CD62L^high^ CD44^low^ T cells was evident (Figure 3A). This phenotype is consistent with that of T_SCM_. [21] Similarly but more dramatically, levels of CD44 on DKO T cells was more significantly reduced (Figure 3 A and B). CD62L^high^ CD44^low^ T cells were prominently present in DKO pmel-1 T cells (Figure 3A). In addition, the adoptive transferred T cells, regardless of their genotypes, were all Sca-1^high^ (Figure 3C). All activated T cells, such as memory stem cells, memory and effector T cells, express very high levels of Sca-1, whereas naïve T cells express low levels of Scal-1. The fact that DKO T cells bore naïve characteristics (CD62L^high^ CD44^low^) but also expressed high levels of Sca-1 was consistent with them being T_SCM_. These data suggest that T-bet plays an important role in maintaining the effector/central memory phenotype. The increase of CD62L^high^ CD44^low^ population was also consistent with our finding that more TKO T cells were found in the lymph nodes (Figure 2C) because CD62L is important for T cell trafficking to the lymph node. Eomes plays an additional role in promoting effector T cell fate, as DKO T cells had a greater decrease in CD44 levels (Figure 3B). Similar decreases in the CD44^high^ population within DKO T cells were observed in lymph node cells (data not shown).

IFN-γ production of adoptively transferred T cells was also examined. About 85% of the donor WT T cells produced IFN-γ (Figure 3 D and E). The frequency of IFN-γ^+^ cells modestly reduced in EKO T cells (Figure 3 D and E). T-bet deletion resulted in lower frequency of IFN-γ producers (Figure 3 D and E). The combined deficiency of T-bet and Eomes led to the greatest reduction of the frequency of IFN-γ producers. T-bet and Eomes were shown to inhibit IL-17 production in CD8 T cells, and lack of both T-bet and Eomes resulted in exacerbated inflammation in a mouse infectious model. [28] However, although we observed a great reduction of IFN-γ producers, we did not find a significant increase of IL-17-producing CD8 T cells within DKO T cells (Figure 3 D). Thus, the increased tumor growth in mice infused with DKO T cells was not due to alteration of IL-17 production.

### T-bet and Eomes are Required for Maintaining Memory T cells in the Blood and Secondary Lymphoid Organs

**Figure 4:**
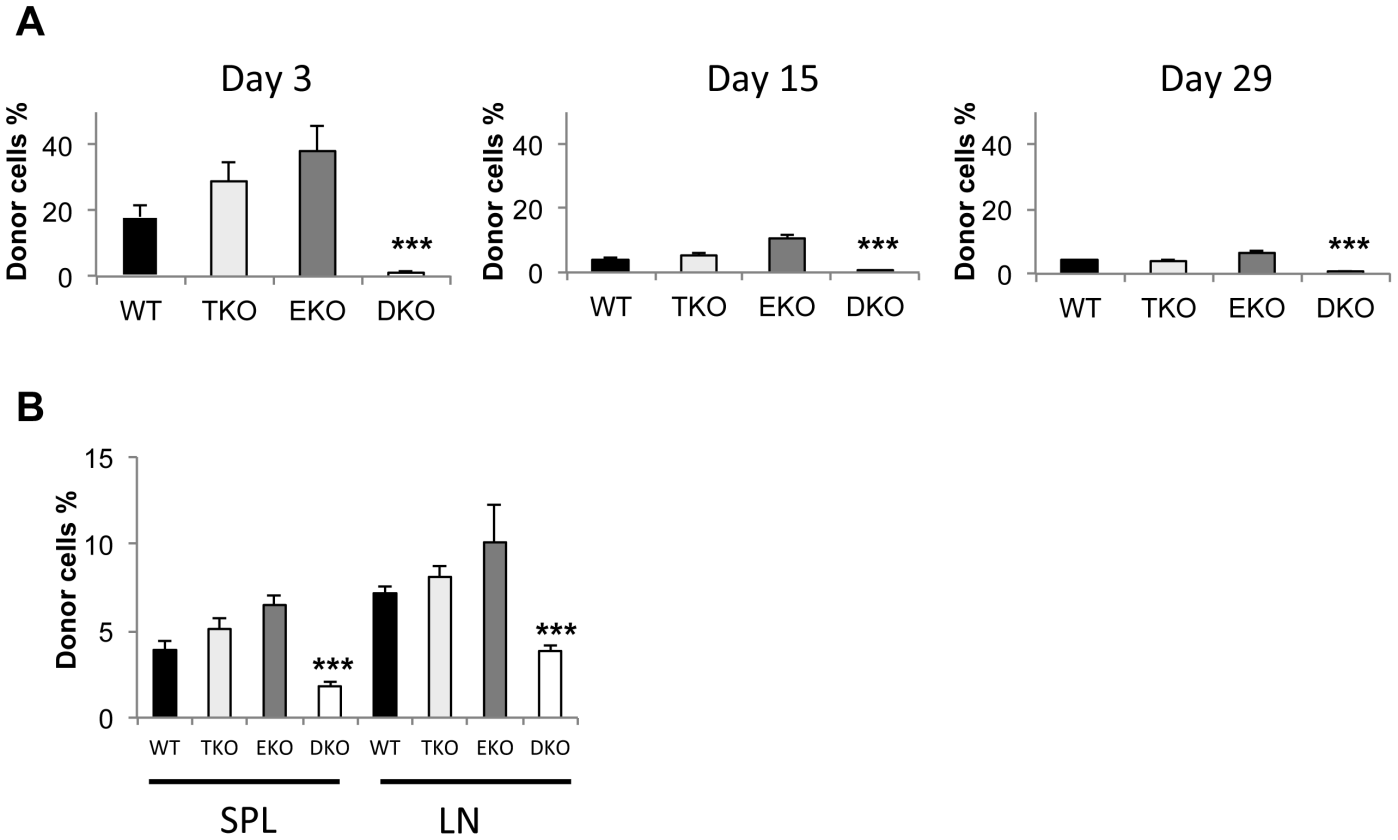
T-bet and Eomes regulate T cell numbers in blood upon adoptive transfer into non-lethally irradiated mice. B6-LY5.2/Cr mice were irradiated at 500 rad. 24 h post irradiation, the mice were adoptively transferred with 5×10^5^ WT, T-bet−/−, Eomes−/−, or T-bet/Eomes DKO pmel-1 T cells which have been cultured in the Th1 condition for 4 days. (A) The recipient mice were bled at different time points and the frequency of the adoptively transferred T cells (CD45.2) among total CD45^+^ cells (CD45.2 plus CD45.1) was determined by flow cytometry. (B) The frequency of the adoptively transferred T cells in spleen and LN were analyzed 30 days post transfer. Data are presented as mean ± SEM of five mice for each genotype. ***P<0.001 by Student’s t-test.

In order to examine the roles of T-bet and Eomes in memory T cells, we infused in vitro-activated WT, TKO, EKO and DKO effector T cells into non-lethally irradiated naïve C57BL/6 mice. We used pmel-1 TCR transgenic T cells, which provide TCR with a strong affinity. This approach allowed tracking of defined populations of antigen-specific memory CD8 cells and avoided the interference of spontaneously generated memory phenotype cells of unknown history. We have included IL-12 in the culture that provided strong inflammatory signals. In addition, the recipient mice had been non-lethally irradiated and conditioned for favorable homeostatic proliferation of the adoptively transferred T cells. All measures ensured a productive generation of memory T cells. [25], [29] Regardless of genotypes, all of the in vitro activated T cells expanded equivalently and were CD44^high^ effector T cells in vitro. These primed T cells were expected to become long-lived memory T cells in vivo upon adoptive transfer. The recipient mice were bled at different time points and the frequency of adoptively transferred T cells was determined by flow cytometry. Three days post adoptive transfer, the frequencies of transferred CD45.2^+^ T cells in the blood were similar among mice receiving WT, TKO or EKO donor T cells (Figure 4A). More than 90% of the CD45.2^+^ cells were CD8^+^Vb13^+^, confirming they were pmel-1 T cells (data not shown). In contrast, the frequency of DKO T cells was significantly lower (Figure 4A). The frequency of infused WT T cells was reduced 15 and 29 days post transfer (Figure 4A). The frequencies of TKO and EKO T cells were similarly reduced and were not different from that of WT T cells. In contrast, the frequency of DKO T cells was drastically reduced in the blood at these time points in comparison to WT T cells (Figure 4A). From day 29 to day 3 post infusion, there were about 4, 7, 6 and 1.5 folds reduction in the percentage of adoptive transferred T cells in the blood for WT, TKO, EKO and DKO T cells respectively. These data suggested that T-bet and Eomes were complementary in determining the number of memory T cells in blood.

Thirty days post transfer, we analyzed the frequency of the adoptively transferred T cells in secondary lymphoid organs. Similar to what we observed in blood, there was no difference in the frequency of memory T cells among the adoptively transferred WT, TKO and EKO T cells in either spleens or lymph nodes. There was about a two-fold and statistically significant reduction of DKO memory T cells compared to WT memory T cells in both spleens and lymph nodes (Figure 4B). The difference between DKO and WT T cells in the blood was far greater than that in the spleen or the in lymph node (Figure 4 A and B). Collectively these data showed that T-bet and Eomes were important in maintaining the memory pool and required for blood circulation of memory T cells.

### T-bet and Eomes were Required for Effector/central Memory T cells but Dispensable for Phenotypic Memory Stem T cells

**Figure 5:**
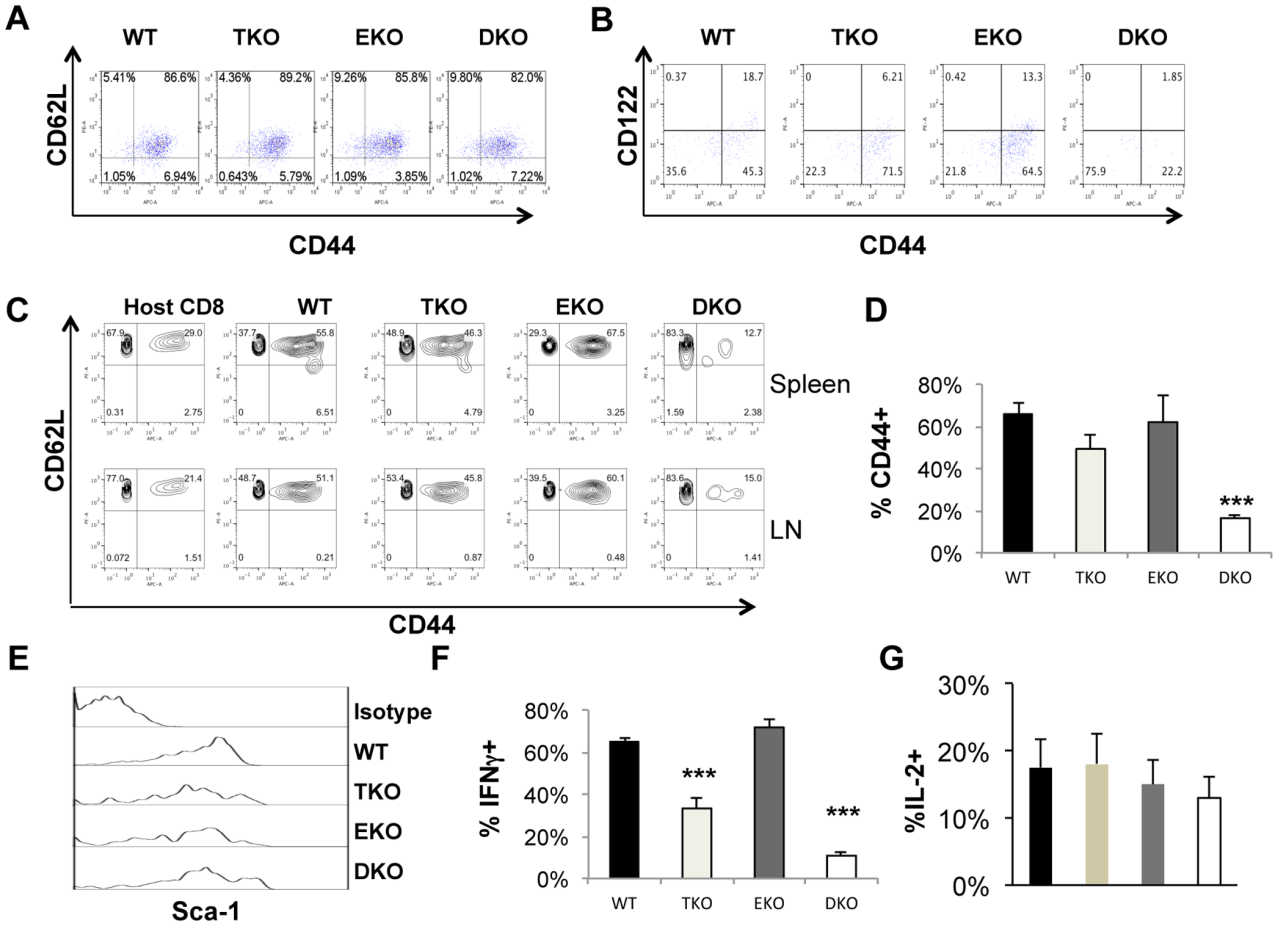
T-bet and Eomes regulate memory subsets of the adoptive transferred T cells. (A) WT, T-bet−/− (TKO), Eomes−/− (EKO), or T-bet/Eomes DKO pmel-1 T cells were cultured in Th1 conditions for 4 days. Expression of CD44 and CD62L on pmel-1 T cells was examined by flow cytometry. (B) B6-LY5.2/Cr mice (CD45.1) were adoptively transferred with WT, T-bet−/−, Eomes−/−, or T-bet/Eomes DKO pmel-1 T cells (CD45.2). Blood was collected from different recipient mice and stained for CD45.2, CD8, CD44, CD122 30 days after transfer. (C) Thirty days post transfer, spleens and lymph nodes (LN) were harvested. Surface markers CD44 and CD62L were examined by flow cytometry. Host cells were CD8^+^CD45.1^+^. WT, TKO, EKO, and DKO indicate representative plots of CD44 versus CD62L of CD8^+^CD4.5.2^+^ donor CD8 T cells. (D) Thirty days post transfer, spleens were harvested. The average percentage of donor cells (CD45.2^+^) CD44^+^ CD8^+^ T cells among all the donor CD8^+^ T cells in the spleen was shown (N = 5 for each genotype). (E) Thirty days post transfer, spleens were harvested. Sca-1 expression on donor CD45.2^+^ CD8^+^ T cells was shown (representative of five independent experiments). (F) and (G) Splenocytes were then stimulated with PMA and ionomycin for 4 hours and incubated for the last 1 hour with Brefeldin A. CD45.2^+^ Cells producing IFN-γ (F) and IL-2 (G) were examined with intracellular cytokine staining and flow cytometry. The average percentages of IFN-γ^+^ and IL-2^+^ cells among the donor CD45.2^+^ CD8^+^ T cells in the spleen were shown (N = 5 for each genotype). Data are presented as mean ± SEM of five samples for each genotype. ***P<0.001 by Student’s t-test.

Memory T cells have been divided into central and effector memory T cells both of which are CD44^high^. Recently, another subset of T cells called stem memory T cells have been discovered which are CD44^low^CD62L^high^. [21], [30] We showed that the level of CD44 on fully activated effector DKO T cells was largely equivalent to that of WT effector T cells after four-day culture (Figure 5A). We examined the surface markers on these cells 30 days after infusion. Because recipient mice had been non-lethally irradiated before T cell infusion, the donor T cells were expanded in the lymphopenic hosts. IL-15 is shown to regulate memory T cell proliferation and previous studies also demonstrated that CD122, the receptor shared by IL-2 and IL-15, is regulated by T-bet and Eomes in naïve mice. [10] Consistent with published results, CD122 expression was partially reduced in both TKO and EKO T cells compared to WT T cells (Figure 5B). In contrast, expression of CD122 was fully ablated in DKO T cells. More strikingly, whereas 60% of the adoptively transferred WT, T-bet−/− and Eomes−/− T cells were CD44^high^, less than 20% of the DKO T cells were CD44^high^ (Figure 5 B, C and D). In sharp contrast, the percentage of CD44^low^ memory cells was greatly increased in DKO pmel-1 T cells compared with T cells of other genotypes (Figure 5 B, C and D). Therefore T-bet and Eomes are important for the survival of CD44^high^ memory T cells, likely mediated by CD122 signaling by IL-15. In contrast, they are not required for CD44^low^ phenotypic T_SCM_. The majority of the CD44^low^ cells were CD62L^high^ (Figure 5C), consistent with a phenotype of T_SCM_. Sca-1 is considered as an important marker for T_SCM_ in mice [21], [22]. We found that all infused T cells were Sca-1^high^ regardless of their genetic backgrounds (Figure 5E). These data suggest that DKO memory T cells had features of T_SCM_. In addition, DKO T cells expressed high similar levels of bcl-2 compared to WT, EKO and TKO T cells. We then examined the ability of the memory T cells to produce IFN-γ. The frequencies of IFN-γ-producers were similar between WT and EKO memory T cells. In contrast, the frequency of IFN-γ+ cells was significantly reduced in TKO T cells (Figure 5 F). The frequency of IFN-γ+ cells was further reduced in DKO T cells (Figure 5 F). These data suggest that T-bet and Eomes all contribute to the immediate effector function of memory T cells, as measured by IFN-γ production. In contrast, similar percentages of adoptively transferred T cells, regardless of their genotypes, made IL-2 (figure 5G). Although CXCR3 was shown expressed in human T_SCM_, [30] it was not expressed on adoptively transferred DKO T cells (data not shown), consistent with our previously published data. [16].

### T-bet and Eomes were Required for the Antitumor Recall Responses Mediated by Adoptively Transferred T cells

**Figure 6:**
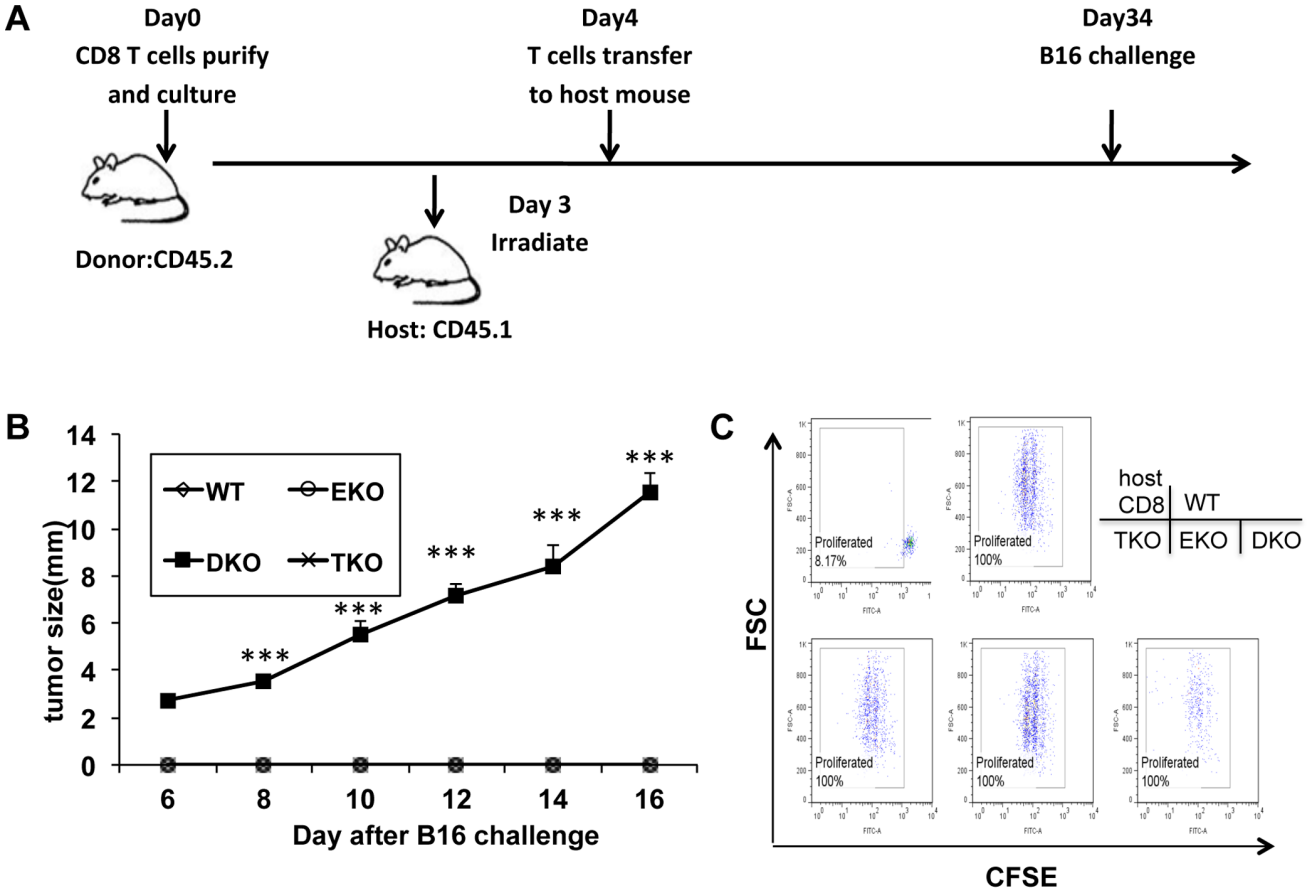
T-bet and Eomes are required for the memory recall responses against tumor cell rechallenge. (A) B6-LY5.2/Cr mice were non-lethally irradiated at 500 rad, within 24 hours of irradiation, mice were adoptively transferred with 4×10^6^ WT, T-bet−/−, Eomes−/−, or T-bet/Eomes DKO pmel-1 T cells which have been cultured in Th1 condition for 4 days. One month later, mice were challenged with 2×10^5^ B16F0 cells *i.d.* (B) Tumor growth was monitored every other day. 5 mice were in each group. ***P<0.001**,** determined by Mann-Whitney Test comparing recipients with DKO and WT T cells. (C) Thirty days post T cell infusion, spleens were harvested. Splenocytes were stained with CFSE and then stimulated with 0.01 µM gp100_25–33_ peptide for 72 h hours. Then the donor cells (CD45.2^+^CD8^+^) were analyzed by flow cytometry for CFSE dilution. Host CD8 T cells were the CD45.1^+^ CD8^+^ population. Data are representative of three independent experiments.

Our data suggest that, despite the drastic reduction of memory T cells in the blood, the total number of memory DKO T cells in the lymph node and spleen was about two-fold reduced compared to WT T cells. The numbers of memory TKO and EKO T cells were similar to that of WT T cells. However, it was not clear whether TKO, EKO or DKO memory T cells were functionally competent. To address this question, we re-challenged recipients with B16 tumor cells and monitored tumor growth (Figure 6 A). The mice infused with WT pmel-1 T cells were fully protected from a second inoculation of tumor cells. Similarly, all mice receiving TKO or EKO pmel-1 T cells were protected from tumor growth (Figure 6 B). In contrast, mice receiving DKO T cells succumbed to the challenge with B16 cells and grew tumor (Figure 6 B). These results suggest that T-bet and Eomes are complementary or redundant in carrying out recall immune responses against tumor.

We then examined whether WT, TKO, EKO, and DKO memory T cells exhibited different proliferative potentials. We re-stimulated the memory T cells from spleens of the recipient mice with 0.01 µM gp100_25–33_ peptide for 72 h in vitro. As expected, host CD8 T cells did not enlarge in size or proliferated (Figure 6C). Without addition of the gp100_25–33_ peptide, the donor T cells also did not proliferate (data not shown). In contrast, the donor WT pmel-1 T cells blasted and proliferated vigorously upon stimulation with the gp100_25–33_ peptide (Figure 6C). This assay also showed no difference in the proliferation potential in recall responses among WT, TKO, EKO, and DKO pmel-1 memory T cells (Figure 6 C). Similar results were obtained with splenocytes stimulated with higher concentrations of gp100_25–33_ peptide (0.1 µM and 1 µM) or with lymph node cells (data not shown). Collectively, these data suggest the effector functions rather than the proliferative potential are controlled by T-bet and Eomes in memory T cells. These results also demonstrated that DKO peml-1 memory T cells were capable of hyper-proliferation upon recall stimulation, even with very low amounts of antigen, consistent with a phenotype of T_SCM_. In addition to proliferation, CD44 could be up-regulated in DKO T cells (figure S3 in file S1), suggesting DKO T cells were capable of some levels of differentiate.

## Discussion

**Figure 7:**
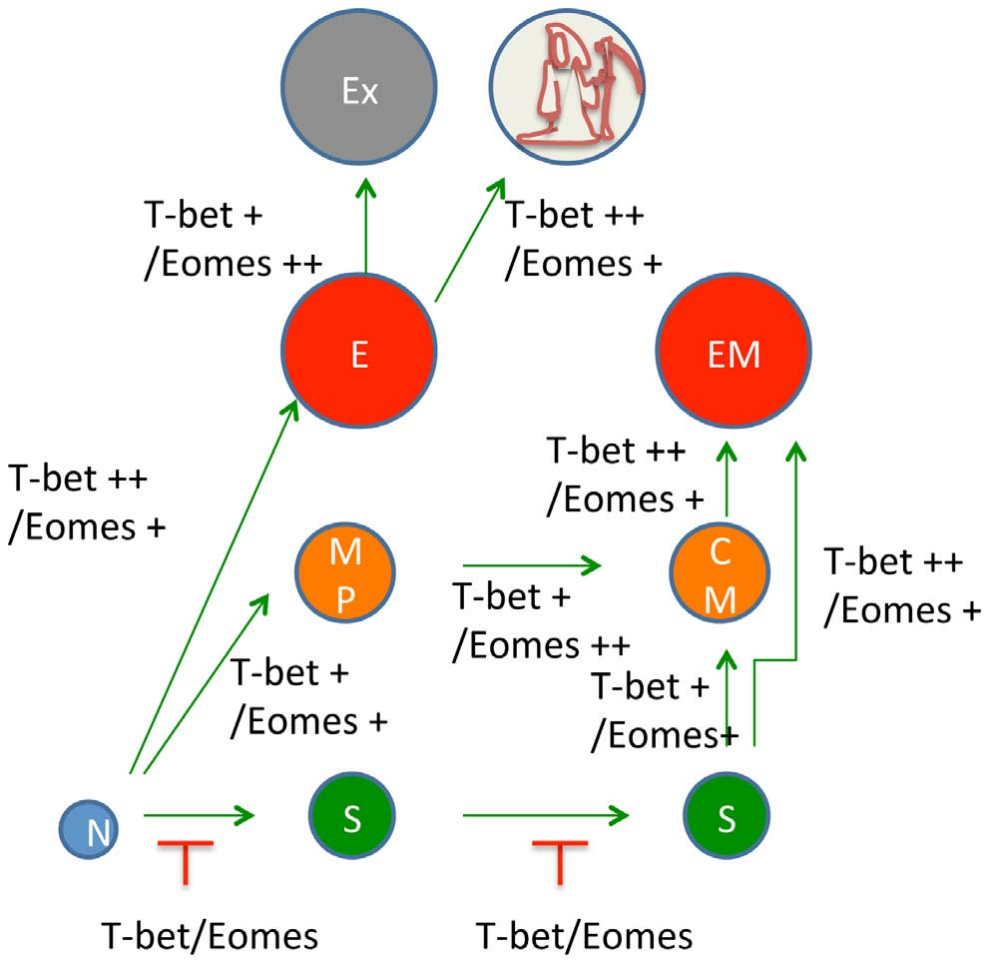
T-bet and Eomes determine memory T cell subsets. Upon activation, naïve T cells are destined to different cellular fates. Current data show that T-bet and Eomes are crucial for the generation of various effector, memory and exhausted T cell subsets. Higher levels of T-bet drive terminally differentiated effector T cells which eventually either become exhausted by further up-regulating Eomes or undergo apoptosis. T cell expressing lower levels of T-bet become memory T cells which also express Eomes. Activated naïve T cells can also take a fate of becoming T_SCM_, which maintain their “naivety” and developmental potential. Our data suggest that T-bet and Eomes regulate the balance between T_EM_, T_CM_, and T_SCM_.

In this study, we examined the role of T-bet and Eomes in both effector and memory responses against tumor. In the setting of adoptive immune therapy of an existing tumor, we showed that the lack of either T-bet or Eomes did not significantly compromise the antitumor effect. However, “knockout” of both of these transcription factors rendered the therapy ineffective. In order to study how T-bet and Eomes regulate the memory T cell pool, we infused T cells of single or double T-bet and Eomes deletion into non-lethally irradiated mice and characterized these T cells and the antitumor recall response. We found that deletion of both T-bet and Eomes in CD8 T cells profoundly inhibited the persistence of effector and central memory T cells. To our surprise, despite the depletion of bulk central and effector memory T cells, the total number of memory DKO T cells was about half of WT T cells in secondary lymphoid organs. Further analysis revealed that deletion of T-bet and Eomes led to a significant increase in the proportion of tumor antigen-specific phenotypic memory stem T cells. These data suggest that T-bet and Eomes are important for differentiation and maintenance of central and effector memory cells, but non-essential or suppressive for generation and persistence of stem cell like memory CD8 T cells (Figure 7).

One interesting outcome of this study is that the DKO memory cells bore characteristics consistent with the phenotype of T_SCM_. [21], [22] The predominant feature of T_SCM_ is CD44^low^ CD62L^hi^ Sca-1^+^. We found majority of DKO memory T cells were indeed CD44^low^ CD62L^hi^ Sca-1^+^. We have also found that before adoptive transfer, all the cells have proliferated and therefore were post-mitotic, fitting another important feature of T_SCM_. [21] In addition, recall response assays showed that DKO memory T cells proliferated similarly upon recall stimulation even with very low antigen. So the recall clonal expansion was normal for DKO memory T cells. Collectively, these data showed that the majority of DKO memory T cells were likely T_SCM_.

Recent studies have firmly established that T_SCM_ have potent antitumor activities upon infusion into host mice [22]. In contrast, DKO T cells, which were phenotypically similar to T_SCM_, were severely compromised in their antitumor function. However these results are not conflicting. We believe the antitumor effect of memory stem cells is dependent on further differentiation of memory stem cells into effector cells in vivo and such differentiation program is dependent on T-bet and Eomes. Although memory stem cells share many properties with central memory T cells, they are thought to be distinctive in terms of developmental stage. Central memory T cells are more differentiated because they express T cell lineage transcription factors such as T-bet and Eomes. In contrast, T_SCM_ are pluripotent and can further differentiate into effector and memory cells. Therefore, the T cell activation program not only results in the generation of effector and central memory T cells with a determined function, but also result in clonal expansion of pluripotent memory stem T cells. Asymmetric cell division is a mechanism that allows both self-renewal and differentiation of stem cells, resulting the production of one stem cell and one differentiating cell. A recent study showing asymmetric segregation of T-bet into two daughter cells after T cell division supports a role of T-bet in dichotomy between differentiated and stem T cells [31]. The benefit of memory stem cells is likely to select and expand T cells with specific activities while preserving their differentiation potential to become effector cells and central memory T cells by taking further inflammatory cues. Our data suggest that T-bet and Eomes regulate the balance between the “stemness” and differentiation of memory T cells (Figure 7).

## Supporting Information

File S1 (PDF)
