# Supplementary material for: T-Bet and Eomes Regulate the Balance between the Effector/Central Memory T Cells versus Memory Stem Like T Cells
Source: PLoS One. 2013 Jun 27;8(6):e67401. doi: 10.1371/journal.pone.0067401 (PMC3694876; doi:10.1371/journal.pone.0067401)
Supplement: File S1 — (PDF) [file pone.0067401.s001.pdf]

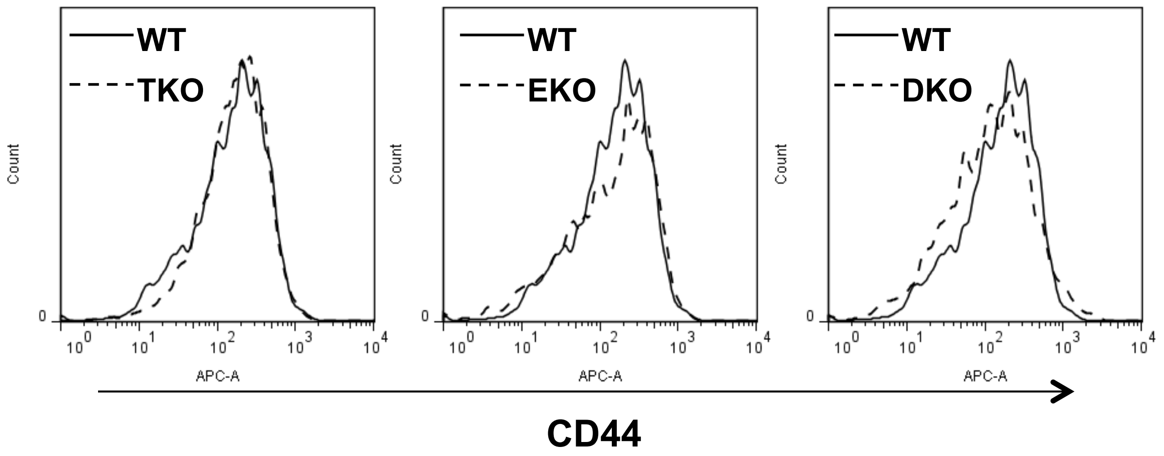

Figure S1. Up-regulation of CD44 on pmel-1 T cells after culture. WT, T-bet-/-, Eomes-/-, or T-bet/Eomes DKO pmel-1 T cells which have been cultured in Th1 conditions for 3 days. The expression of CD44 on these cells was analyzed by flow cytometry. Data represents three independent experiments. One mouse from each strain was used in each experiment.

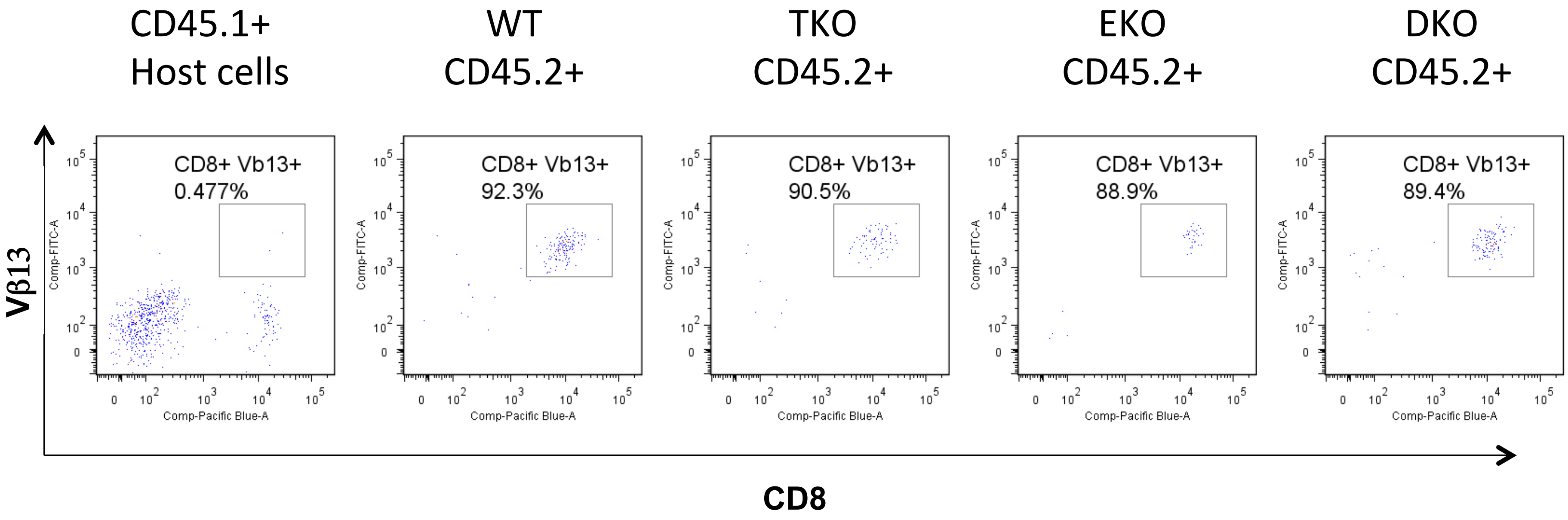

Figure S2. Pmel-1 T cells were the majority of the adoptively transferred cells. B6-LY5.2/Cr mice were challenged with 3×10^5 B16F0 cells *i.d.* 6 days later, mice were irradiated at 500 rad. On day 7, the mice were adoptively transferred with 5 x10^5 WT, T-bet-/-, Eomes-/-, or T-bet/Eomes DKO pmel-1 T cells which had been cultured in Th1 conditions for 3 days. Mice were bled three days after T cell infusion. The percentage $CD8^+$ $V\beta13^+$ (pmel-1) T cells among the donor cells ($CD45.2^+$) cells was determined by flow cytometry and the representative dot plots were shown.

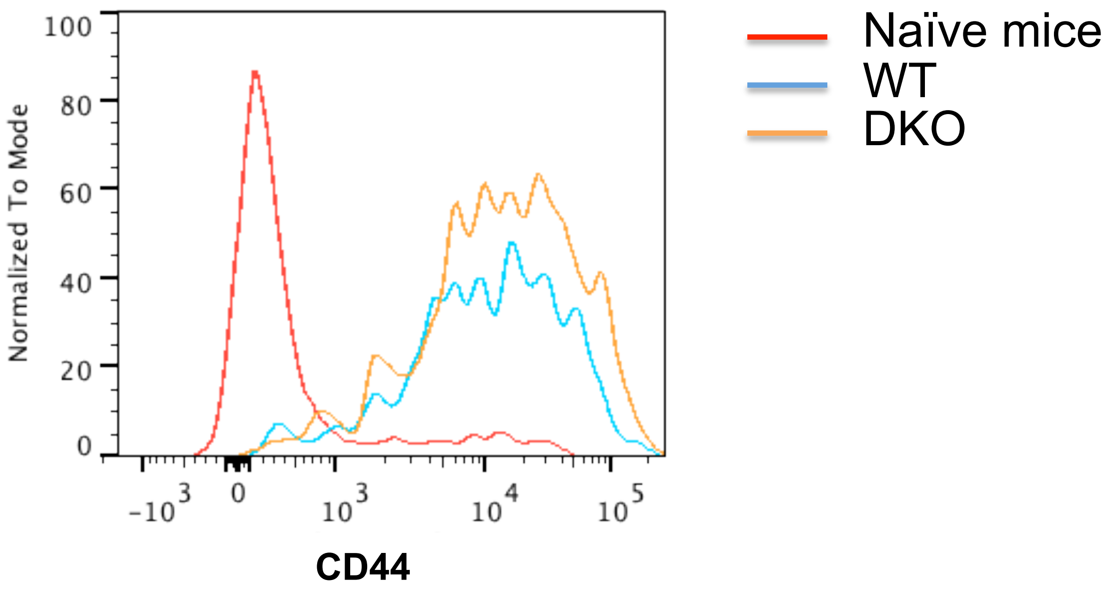

Figure S3. Up-regulation of CD44 on memory pmel-1 T cells after ex vivo re-stimulation. B6-LY5.2/Cr mice were irradiated at 500 rad, within 24 hours of irradiation, mice were adoptively transferred with 4×10^6 pmel-1 T cells (WT and DKO) which have been cultured in Th1 conditions for 4 days. Thirty days post T cell infusion, spleens were harvested. Splenocytes were stimulated with 0.01 mM $gp100_{25–33}$ peptide for 72 h hours. Then the donor cells ($CD45.2^+CD8^+$) were analyzed by flow cytometry for CD44 expression (WT denotes wild type donor cells, DKO denotes T-bet -/- Eomes -/- donor cells, and naïve mice denote CD8 T cells from unmanipulated wild type mice which were used as control.
